# Supplementary figures and images for: A Survey of Main Pepper Crop Viruses in Different Cultivation Systems for the Selection of the Most Appropriate Resistance Genes in Sensitive Local Cultivars in Northern Spain
Source: Plants (Basel). 2022 Mar 8;11(6):719. doi: 10.3390/plants11060719 (PMC8951742; doi:10.3390/plants11060719)

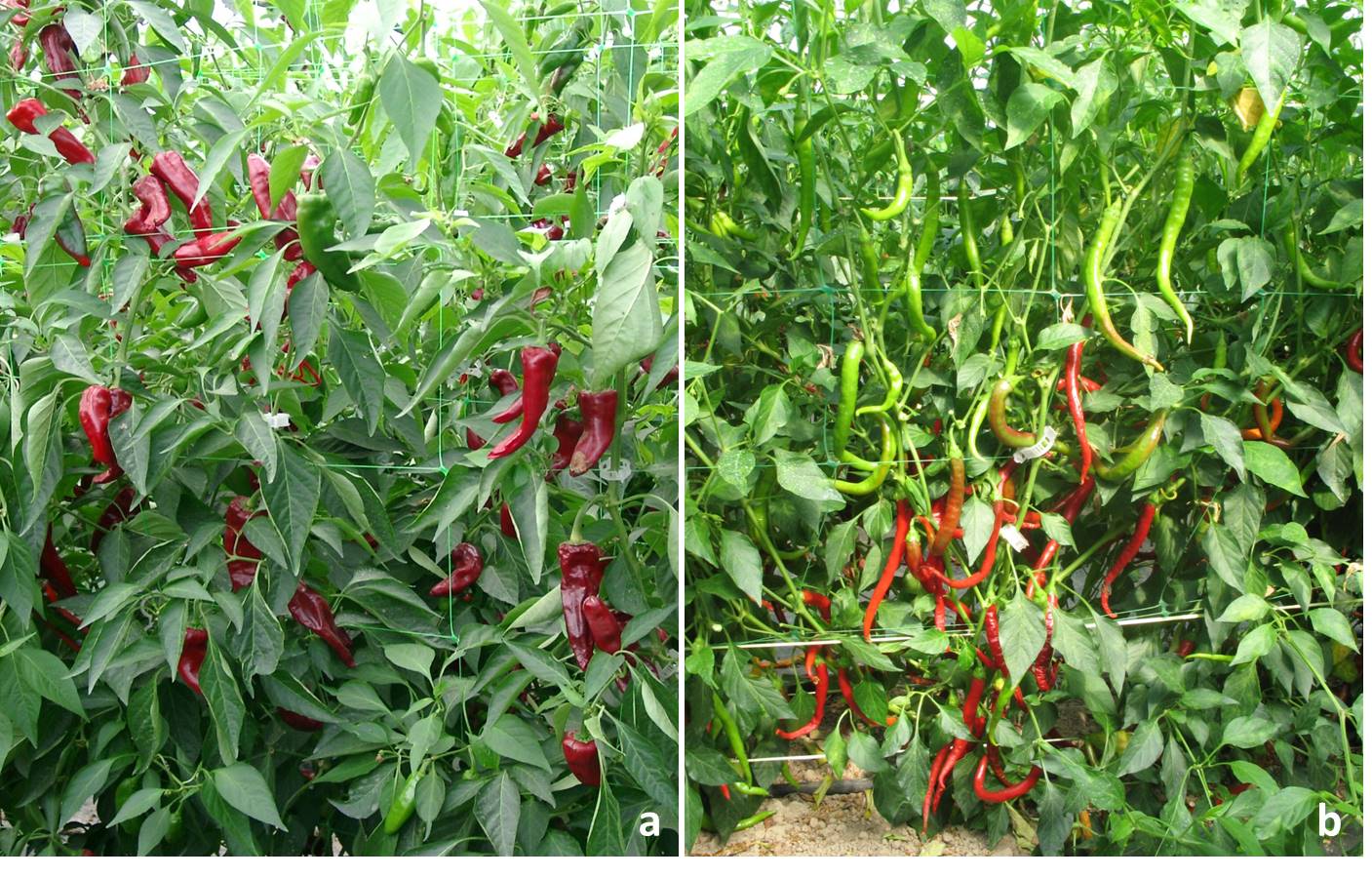

Supplement: Supplementary file 1 [file plants-11-00719-s001.zip › plants-1583721-supplementary/Figure S1.jpg]

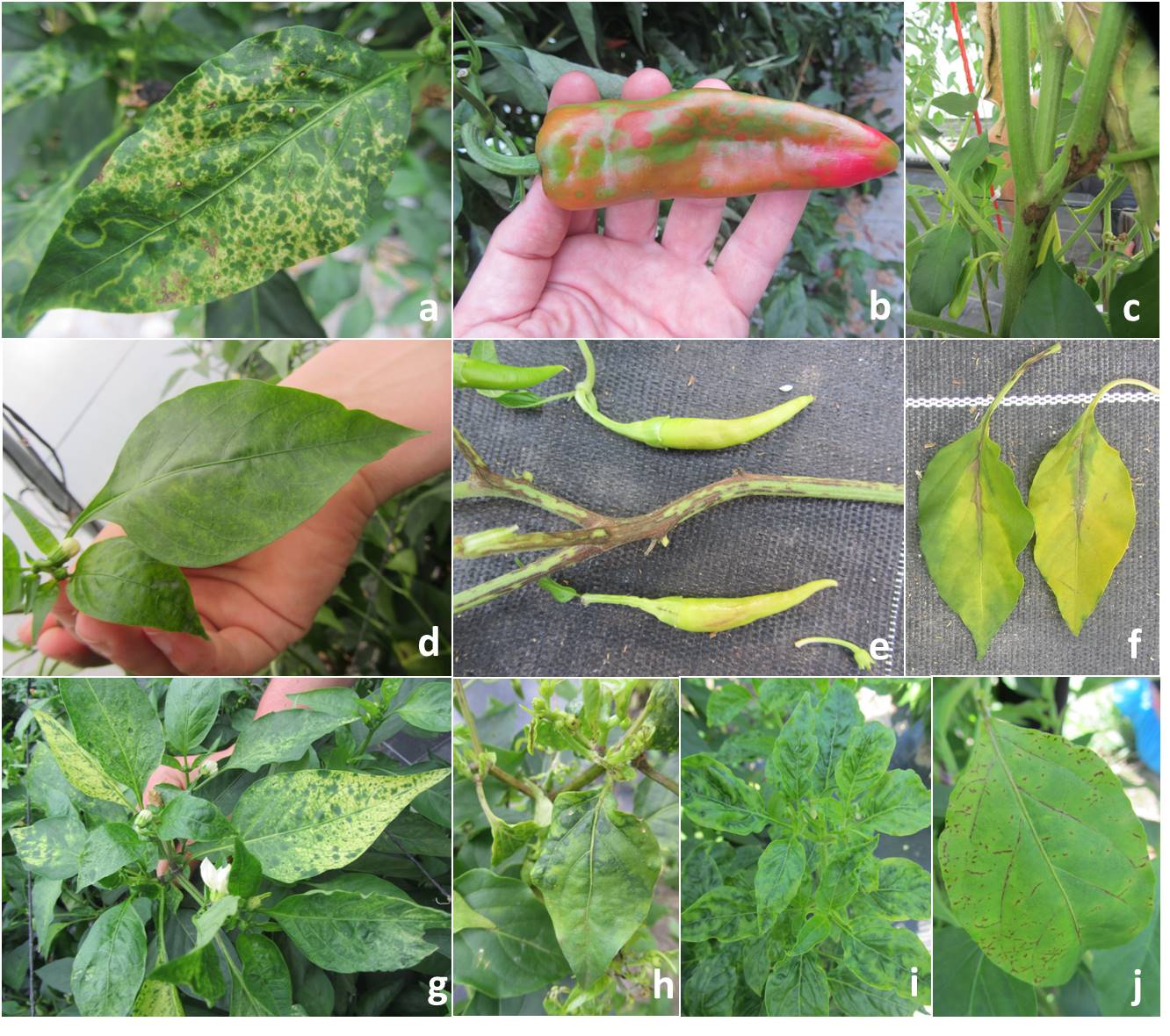

Supplement: Supplementary file 1 [file plants-11-00719-s001.zip › plants-1583721-supplementary/Figure S2.jpg]

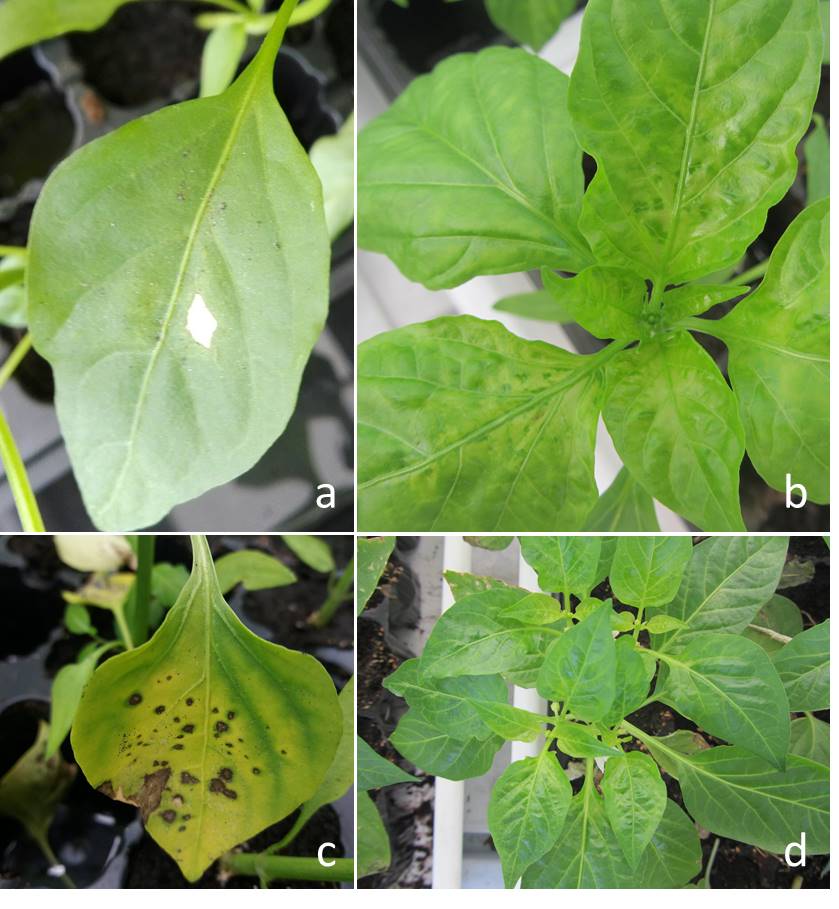

Supplement: Supplementary file 1 [file plants-11-00719-s001.zip › plants-1583721-supplementary/Figure S3.jpg]

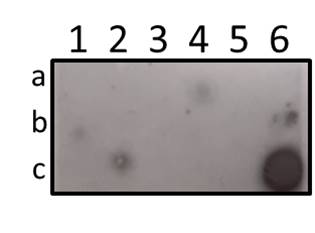

Supplement: Supplementary file 1 [file plants-11-00719-s001.zip › plants-1583721-supplementary/Figure S4.jpg]
